# Modeling Dominant and Recessive Forms of Retinitis Pigmentosa by Editing Three *Rhodopsin*-Encoding Genes in *Xenopus Laevis* Using Crispr/Cas9

**DOI:** 10.1038/s41598-017-07153-4

**Published:** 2017-07-31

**Authors:** Joanna M. Feehan, Colette N. Chiu, Paloma Stanar, Beatrice M. Tam, Sheikh N. Ahmed, Orson L. Moritz

**Affiliations:** 1 Department of Ophthalmology and Visual Sciences, University of British Columbia, Vancouver, British Columbia Canada V5Z 3N9; 2 The Sainsbury Laboratory, Colney Ln, Norwich Research Park, Norwich Norfolk, UK NR4 7UH

## Abstract

The utility of *Xenopus laevis*, a common research subject for developmental biology, retinal physiology, cell biology, and other investigations, has been limited by lack of a robust gene knockout or knock-down technology. Here we describe manipulation of the *X. laevis* genome using CRISPR/Cas9 to model the human disorder retinitis pigmentosa, and to introduce point mutations or exogenous DNA sequences. We introduced and characterized in-frame and out-of-frame insertions and deletions in three genes encoding *rhodopsin* by co-injection of Cas9 mRNA, eGFP mRNA, and single guide RNAs into fertilized eggs. Deletions were characterized by direct sequencing and cloning; phenotypes were assessed by assays of rod opsin in retinal extracts, and confocal microscopy of cryosectioned and immunolabeled contralateral eyes. We obtained germline transmission of editing to F1 offspring. In-frame deletions frequently caused dominant retinal degeneration associated with rhodopsin biosynthesis defects, while frameshift phenotypes were consistent with knockout. We inserted eGFP or point mutations into *rhodopsin* genes by co-injection of repair fragments with homology to the Cas9 target sites. Our techniques can produce high frequency gene editing in *X. laevis*, permitting analysis in the F0 generation, and advancing the utility of *X. laevis* as a subject for biological research and disease modeling.

## Introduction

The African clawed frog *Xenopus laevis* is an amphibian species often used in laboratory research due to a number of desirable properties, including induction of breeding by hormone injection, minimal housing requirements, and no requirement for live food^1, 2^. It is frequently used in studies of developmental biology, cell biology, retinal research, electrophysiology, and numerous other applications. In our laboratory we make use of *X. laevis* in cell biology studies of retinal photoreceptors^3–6^, including modeling of the human disorder retinitis pigmentosa^7–11^, due to the relative ease with which *X. laevis* can be genetically modified by transgene insertion. Non-chimeric transgenic *X. laevis* can be generated using the method of Kroll and Amaya^12^ or other methodologies^13–15^ at very high rates. F0 animals can be analyzed rather than waiting for F1 offspring, allowing very short experimental timeframes^16^.

However, robust knockout and knockdown technologies for *X. laevis* have not been previously available. Although morpholinos can be used in early developmental stages^17–19^ it is difficult to discriminate non-specific effects^20^. *X. laevis* do not have robust RNAi responses^21^, and techniques analogous to standard mouse knockout procedures requiring embryonic stem cells are not feasible due to lack of cell lines. A further complication is the allotetraploid nature of the *X. laevis* genome^22–24^.

Recent development of the CRISPR/Cas9 gene editing system should allow gene modification and knockout in virtually any species, including *X. laevis* ^25^. This two component system consists of a DNA-cleaving enzyme (Cas9) complexed with an RNA that guides Cas9 to a specific DNA sequence (single guide RNA or sgRNA)^26–28^. Cleavage triggers the non-homologous end-joining (NHEJ) DNA repair mechanism, frequently resulting in insertion or deletion of short DNA sequences (indels). No other endogenous factors are required. Indels in early exons can create frame-shift mutations that trigger nonsense mediated decay, acting as loss-of function mutations or “knockouts”, or in-frame deletions and insertions that result in protein instability. This technology has previously been successfully applied to generate knockouts of tyrosinase in *X. laevis* and the related diploid species *Xenopus tropicalis* ^29, 30^ to generate F0 albino animals. Additionally, breaks in genomic DNA caused by Cas9 can trigger homology-directed repair (HDR) which can be used to introduce DNA sequences at specific sites via homologous recombination^31, 32^.

Here we report the successful application of CRISPR/Cas9 technology to editing of the *X. laevis* genome. As a target, we chose the genes encoding *X. laevis* rhodopsin (i.e. rod opsin). Rhodopsin is expressed at very high levels exclusively in retinal rod photoreceptors, and is a focus of our research program. Based on previous reports of *rhodopsin* knockouts in mice^33, 34^, loss-of-function *rhodopsin* mutations in humans, and missense *rhodopsin* mutations in humans and a variety of transgenic animals, we anticipated that CRISPR/Cas9 based gene editing of the *rhodopsin* genes would cause non-lethal phenotypes in *X. laevis*. We anticipated that loss-of-function mutations would cause reduced rhodopsin expression, while mutations that alter the amino acid sequence may cause aggressive RD, and that these phenotypes would be detectable by rhodopsin immunoassays and confocal microscopy^7^.

By using this technology to create genomic DNA breakpoints and indel mutations, we were able to generate *rhodopsin* knockout and gain of function phenotypes in *X. laevis* tadpoles that model the human conditions of recessive and dominant retinitis pigmentosa. Furthermore, we were able to use the technology to selectively edit specific alleles, and to introduce foreign DNA sequences and point mutations by homologous recombination. Genome editing occurred in germline cells, allowing efficient passage of phenotypes to F1 generation tadpoles. These techniques add new versatility to *X. laevis* as a research subject, and are likely to be highly useful for cell and developmental biologists, including those conducting research on rhodopsin function and *rhodopsin* gene regulation, and retinal disease mechanisms.

## Materials and Methods

### *In vitro* transcription of mRNA and sgRNA, and sgRNA design

Cas9 mRNA was *in vitro* transcribed from pMLM3613 (A gift from Keith Joung - Addgene plasmid #42251), linearized using PmeI,, with T7 mMessage mMachine Ultra kit (Ambion) or HiScribe ARCA kit (NEB) according to the manufacturer’s instructions and subsequently purified using the RNeasy kit (Qiagen). eGFP mRNA was *in vitro* transcribed from a linear plasmid template using the T7 mMessage mMachine Ultra kit (Ambion) according to the manufacturer’s instructions and subsequently purified using the RNeasy kit (Qiagen).

sgRNA target sites were identified by scanning published *X. laevis* rhodopsin sequences using the online ZiFiT target finding tool (http://zifit.partners.org/ZiFiT/). Three sgRNAs were designed and tested: rhosg3 (targets exon 1 of *rho.2.L*), rhosg1 (targets exon 1 of all *X. laevis* rhodopsin genes), and rhosg4 (targets the final exon 5 of all *X. laevis* rhodopsin genes). See Supplementary information Fig. 1 for details.

Oligonucleotides corresponding to these sgRNA targeting sequences were cloned into the Bbs1 site of pDR274 (A gift from Keith Joung - Addgene plasmid #42250). This plasmid contains a sgRNA scaffold. The pDR274 derivatives were linearized with Dra1 and used as templates for *in vitro* transcription of sgRNA using the MAXIscript *in vitro* transcription kit (Ambion) and subsequently purified using miRNeasy kit (Qiagen). Two individual reactions were then combined and concentrated by ethanol precipitation. pMLM3613 and pDR274 were previously used for generating Cas9 mRNA and sgRNAs for use in zebrafish genome editing^35^.

Final products were evaluated for size and quality by agarose gel electrophoresis, and quantified by absorbance at 260 nm with NanoDrop 2000c spectrophotometer (Thermo Scientific).

### Microinjections

Ovulation was induced in female *X. laevis* by injection of human chorionic gonadotropin, and testes were isolated from male *X. laevis*. One-half of one testis was macerated in 250 µL 0.1X MMR and then added to eggs manually expelled into a petri dish. After a 2-minute incubation, the eggs were covered in 0.1X MMR and incubated a further 20 minutes. The fertilized eggs were then dejellied in 2% cysteine pH 8.0, 1X MMR, and arranged in a monolayer covered with 0.4X MMR, 6% Ficoll. RNAs were combined, centrifuged at 13,000 RPM for 1 minute prior to loading into a pulled glass micropipette with a 20–25 µm bore which was then connected to a Hamilton syringe pump set to deliver 36 µL per hour, and mounted in a micromanipulator. Embryos were injected for 1 second equating to 10 nL of RNA. After microinjection, embryos were transferred to 18 °C for approximately 2.5 hours until the four-cell stage, at which point embryos that exhibited appropriate signs of cell division were transferred to 0.1X MMR + 6% Ficoll +10 µg/mL gentamicin and stored overnight at 18 °C. Embryos were transferred to 0.1X MMR 24 hrs post-fertilization. Embryos were screened for eGFP fluorescence using an epifluorescence-equipped Leica MZ16F dissecting microscope.

### PCR products/cloning of PCR products

For analysis of indels, approximately 300 bp of sequence around the rhosg1 and rhosg3 target-sites were amplified by PCR using primer sequences: CAGTTGGGATCACAGGCTTC and CAGGATGTAGTTTAGGGGTG for *rhodopsin* chromosome 4L paralogs *rho.L* and *rho.2.L*, and primer sequences: CACAGAAGGCATTCTTTCTAG and CAGCAAGATGTAGTTTAAGGGTG for the *rho.S* homeolog. Two *rho.S* forward primers were used due to the presence of two alleles in database files.

### Direct sequencing of PCR products and sequencing of clones

PCR products were directly sequenced with the respective forward primers by the Sanger dye-termination method after exonuclease I-shrimp alkaline phosphatase treatment^36^. PCR products were ligated into EcoRV-linearized pBluescript-SKII+ by a directional Gibson assembly reaction^37^, and used to transform *E. coli*, allowing isolation of individual genomic sequence clones. For cloning, we utilized loci-specific primers with sequences identical to those above, with additional 24 base pair “tails” with homology to pBS-SKII+ required for the directional Gibson assembly reaction. Forward primers incorporated the 5′ homology tail sequence GTCGACGGTATCGATAAGCTTGAT, while reverse primers incorporated the 5′ homology tail sequence TCCCCCGGGCTGCAGGAATTCGAT. Overnight cultures derived from individual colonies were mini-prepped and sequenced by the Sanger dye-termination method using M13 universal primer.

### Determining extent of genomic editing

To provide a measure of the extent of genome editing that could be compared between samples, we developed an algorithm to summarize the deviation from wildtype (WT) sequence at each nucleotide position of a direct sequencing reaction read as a “sequence fidelity score”. Sanger sequencing data output files (generated as *.abi files by the ABI Prizm Sanger sequencing software) were uploaded to the Life Technologies ab1PeakReporter online tool (https://apps.thermofisher.com/ab1peakreporter/), which extracts information regarding primary (most probable) and secondary (second most probable) base calls (largely based on the highest and second-highest peaks at each nucleotide position) into a file readable by Microsoft Excel (http://tools.thermofisher.com/content/sfs/brochures/seq-quantification-app-note.pdf). We identified the primary and secondary peak base call for each nucleotide position in sequencing reads from experimental samples using the ab1PeakReporter tool, and assigned a numerical value for each position of the experimental sequencing read based on comparison to the primary base call in an aligned control WT sequencing read. A perfect match of the primary base call with WT was assigned a score of 1, and a mismatched primary base but matched secondary base was assigned a score of 0.25. Complete mismatch was assigned a score of zero. In addition, partial matches with ambiguous base calls such as “Y” and “R”, as defined by the International Union of Pure and Applied Chemistry were assigned intermediate scores. The complete scoring formula is shown in Supplemental Table 1. For comparison plots, the scores were plotted as moving averages over 10 bases.

### Dot blot assay for rod opsin

Individual tadpole eyes were solubilized, and aliquots were diluted and applied to Immobilon P membranes (Millipore) as previously described^7^. Blots were probed with anti-rhodopsin antibody B630N and IR-dye800 secondary antibody (LI-COR), and imaged and quantified using a LI-COR Odyssey imaging system as previously described^7^.

### Confocal microscopy

Eyes were fixed overnight at 4 °C in 4% paraformaldehyde in 0.1 M phosphate buffer pH 7.4, and then transferred to 20% sucrose in phosphate buffer pH 7.4 at 4 °C for one hour before embedding and quick-freezing in OCT medium (TissueTek). Eyes were cryosectioned (12 µm). Cryosections were collected on Superfrost glass slides (Fisher Scientific) and immunolabeled for confocal microscopy. Sections were soaked in phosphate buffered saline pH 7.4 (PBS), and blocked in PBS containing 1% goat serum and 0.1% TX-100 for 20 minutes, then rinsed 3X in PBS, and incubated with primary antibodies overnight in PBS containing 0.1% goat serum and 0.1% TX-100. Antibodies and dilutions used were anti-rhodopsin monoclonal antibodies B630N and 514–18 (tissue culture supernatants used at 1:20 dilution), anti-mammalian rhodopsin antibody 2B2 (tissue culture supernatant used at 1:10 dilution) and polyclonal anti-rod alpha-transducin (1:1000 dilution). Primary antibodies were washed off in 3 changes of PBS, and secondary antibodies were applied diluted in the same buffer for four hours. Secondary antibodies were Cy3-conjugated anti-mouse and Alexa Fluor 488 anti-rabbit (Jackson) (1:750 dilution). Counterstains of Alexa Fluor 647 wheat germ agglutinin (WGA, 5 µg/ml, Life Technologies) (labels Golgi, plasma membrane, and outer segment membranes) and Hoechst 33342 nuclear stain (0.1 mg/ml, Sigma-Aldrich) were included in the secondary antibody labeling step. Slides were rinsed in 3 changes of PBS, cover slipped using Mowiol mounting medium (Sigma Aldrich), and imaged using a Zeiss 510 confocal microscope equipped with a 40X N.A. 1.2 water immersion objective. Antibodies B630N, 514–18, and anti-rod alpha-transducin were gifts from W. C. Smith. Antibody 2B2 was a gift from R.S. Molday.

### Electron microscopy

Animals were fixed in 1% glutaraldehyde, 4% paraformaldehyde in 0.1 M phosphate buffer pH 7.4. Cryosections were obtained, embedded in Eponate12 resin, and processed for electron microscopy using procedures identical to those previously described^38^.

### HDR procedures

HDR repair fragments were ligated into EcoRV-linearized pBluescript-SKII+ by Gibson assembly^37^. *Rhodopsin* fragments were amplified from genomic DNA using primers based on the *rhodopsin* gene sequence published by Batni *et al*.^39^ (corresponding to *rho.2.L*), and eGFP sequences were amplified from eGFP-C1 (Clontech). The sgRNA target-sites were subsequently mutagenized and the M13F epitope inserted using the quickchange mutagenesis kit (Agilent). Constructs were purified using the Miniprep Kit (Qiagen), linearized at SmaI, and concentrated using QIAEX II bead purification kit (Qiagen). The eyes of eGFP-HDR animals were screened for fluorescence using an epifluorescence-equipped dissecting microscope.

### Animal care

Experimental procedures were approved by the UBC Animal Care Committee and were performed in accordance with Canadian Council on Animal Care guidelines and regulations.

## Results

### Rhodopsin gene organization in *X. laevis*

We searched genomic DNA sequence data from the *X. laevis* genome project available at Xenbase^40^ for sequences encoding rod opsins. Based on blast searches using previously published *X. laevis rhodopsin* gene sequence^39^, we identified three gene sequences encoding rod opsins, i.e. rhodopsin, *rho.S* (XB-GENE-17342665) on chromosome 4S, and two paralogous gene sequences, *rho.L* (XB-GENE-966893) and *rho.2.L* (XB-GENE-18034123), corresponding to the sequence identified by Batni *et al*.^39^ located within a 17 kb region on chromosome 4 L. These gene sequences have highly similar coding regions (Supplementary Figure 1), although intron regions have diverged considerably. Matching EST sequences present in the Sanger Centre databases indicate that all three genes are transcriptionally active. All differences between paralogs and homeologs in coding regions are either silent or result in conservative amino acid changes, suggesting all three genes encode functional rhodopsins.

### Injected Cas9 mRNA is not significantly toxic to *in-vitro* fertilized embryos

Based on Cas9 mRNA quantities utilized by Nakayama *et al*.^41^ for genome editing in *X. tropicalis* ^29^, we compared the survival rates of *in vitro* fertilized embryos injected with 14 ng, 5 ng, 1.5 ng, 0.5 ng, or 0.15 ng of Cas9 mRNA at the single-cell stage. 700 pg of eGFP mRNA was co-injected as a reporter for successful Cas9 mRNA delivery. Toxicity was not evident in any of the injection groups, as survival rates of GFP-expressing embryos were not different from uninjected embryos. At 14 days post-fertilization (dpf) (Nieuwkoop and Faber developmental stage 48^42^), morphology of tadpoles derived from injected embryos was not different than morphology of tadpoles derived from wildtype (WT) uninjected embryos (not shown).

### Injection of Cas9 mRNA+ guide RNA induces retinal degeneration phenotypes

**Figure 1:**
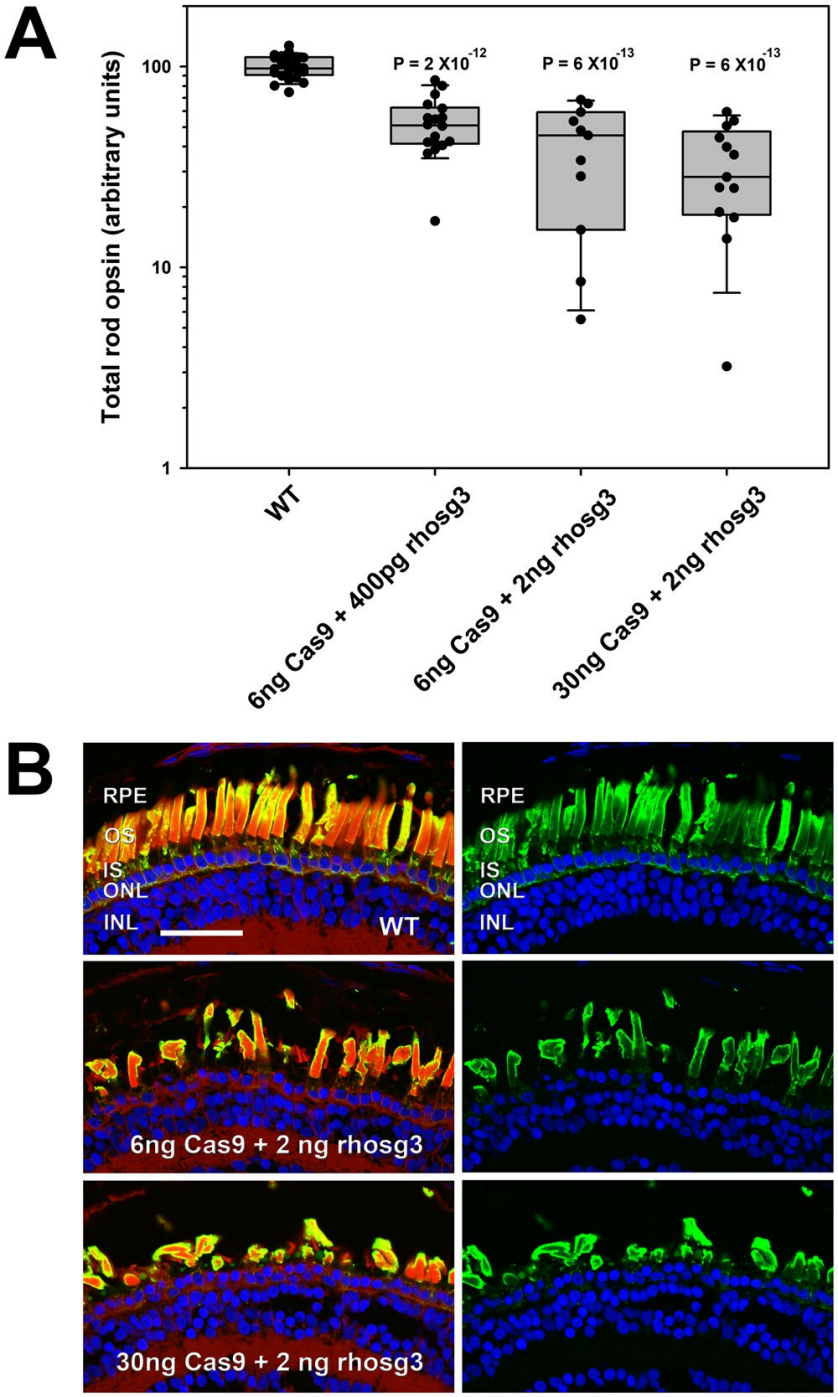
Injection of Cas9 and guide RNAs causes reduced rod opsin levels and retinal degeneration. (**A**) Rod opsin levels assayed in individual eyes by dot blot assay at 14 dpf. Each data point represents a different animal. P values are for Tukey multiple comparisons test for comparisons with WT group. P (ANOVA) = 4.1 × 10^−19^ (**B**) Phenotypes assessed by confocal microscopy. Retinas from animals injected with Cas9 mRNA and rhosg3 had missing and malformed rod photoreceptors. Green: anti-rhodopsin (B630N). Red: wheat germ agglutinin. Blue: Hoechst 33342. RPE: retinal pigment epithelium. OS: outer segments. IS: inner segments. ONL: outer nuclear layer. INL: inner nuclear layer. Bar = 50 µm.

We designed single guide RNAs (sgRNAs) that target the first exon of *rho.S*, *rho.L* and *rho.2.L* and injected varying concentrations of Cas9 mRNA and sgRNA, along with eGFP mRNA, into fertilized *X. laevis* eggs. Non-fluorescent, weakly or partly fluorescent embryos were eliminated from the analysis at 1 dpf. At 14 dpf, we analyzed the resulting tadpole phenotypes by anti-rod opsin dot blot assay of total eye protein extracts and confocal microscopy of contralateral eyes as previously described^7^. Our initial experiments were conducted before we were aware of the presence of the *rho.2.L* paralog, which we identified after the release of the *X. laevis* genome (http://www.nature.com/nature/journal/v538/n7625/abs/nature19840.html). These experiments utilized rhosg3, an sgRNA whose target sequence is an exact match only with *rho.L*. We noted increasingly severe phenotypes of reduced rod opsin protein levels (Fig. 1A) and RD (Fig. 1B) on injection of higher concentrations of both sgRNA and Cas9 mRNA (Fig. 1), consistent with disruption of *rhodopsin* genes. However, no animals with complete absence of rod opsin were generated.

### rhosg1 generates more severe phenotypes than rhosg3

**Figure 2:**
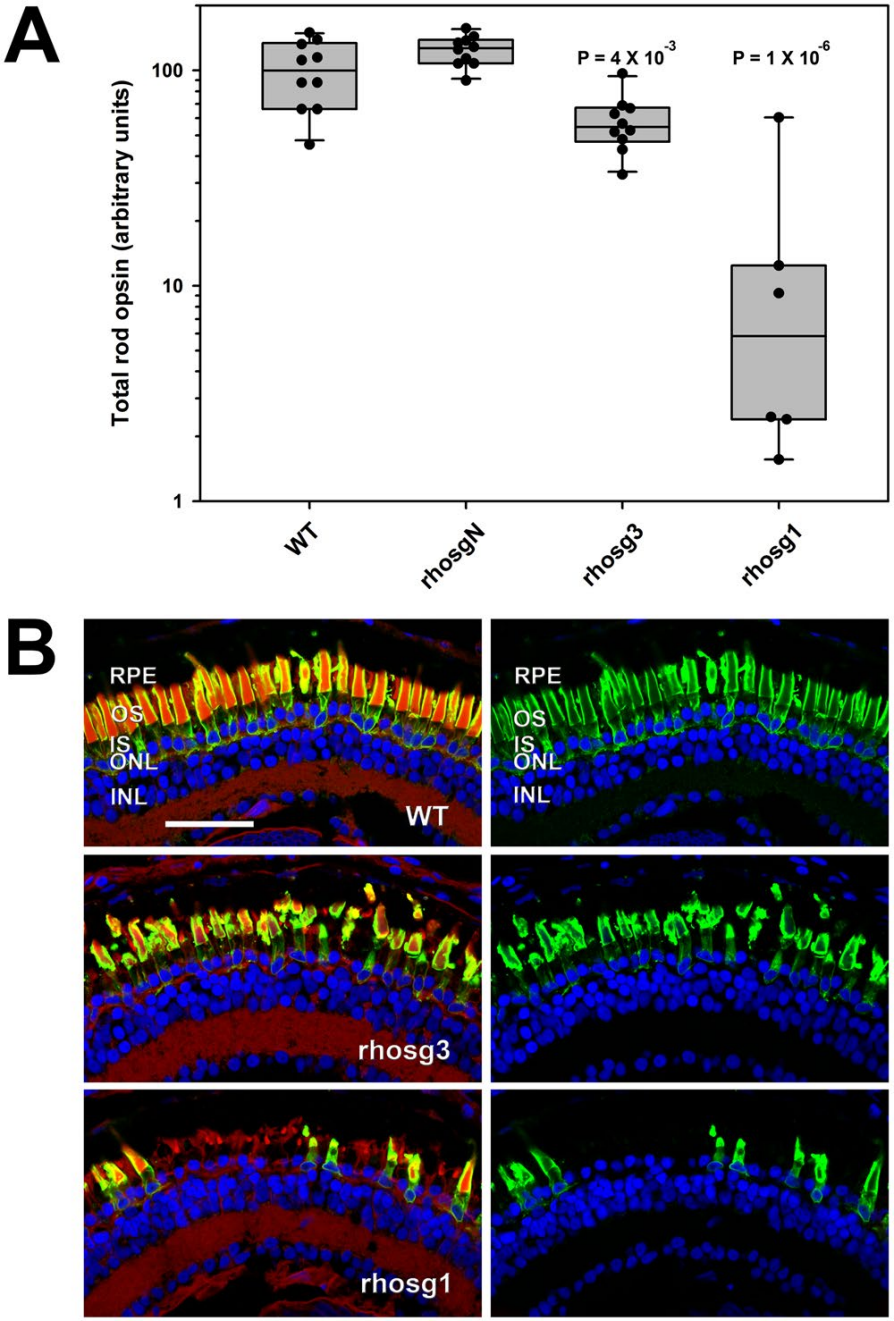
Relative to guide RNA rhosg3, rhosg1 causes greater reductions in rod opsin and greater rod photoreceptor loss. 6 ng of Cas9 mRNA and 2 ng of sgRNA were co-injected. (**A**) Rod opsin levels assayed in individual eyes by dot blot assay at 14 dpf. Each data point represents a different animal. P values are for Tukey multiple comparisons test for comparisons with WT group. P (ANOVA) = 2.9 × 10^−9^ (**B**) Phenotypes assessed by confocal microscopy. Green: anti-rhodopsin (B630N). Red: wheat germ agglutinin. Blue: Hoechst 33342. RPE: retinal pigment epithelium. OS: outer segments. IS: inner segments. ONL: outer nuclear layer. INL: inner nuclear layer. Bar = 50 µm.

*rho.S* and *rho.2.L* are not targeted by the sgRNA rhosg3 (used in the experiment shown in Fig. 1) due to the presence of two mismatched bases at positions 1 and 6 in the protospacer. We therefore compared the effects of rhosg3 with those of a second sgRNA (rhosg1) that targets all gene variants identified (Supplementary Figure 1), as well as a control non-targeting sgRNA (rhosgN, the reversed sequence of rhosg3). We observed substantially greater reduction in total rod opsin in 14 dpf animals on co-injection of Cas9 mRNA+ rhosg1 in comparison to Cas9 mRNA+ rhosg3 (Fig. 2). Again, complete absence of expression was not observed, but total rod opsin levels averaged 7% of WT. In contrast, minimal effects on total rod opsin were observed on co-injection of Cas9 mRNA+ rhosgN.

### Indels are present in the genomic DNA of F0 animals

**Figure 3:**
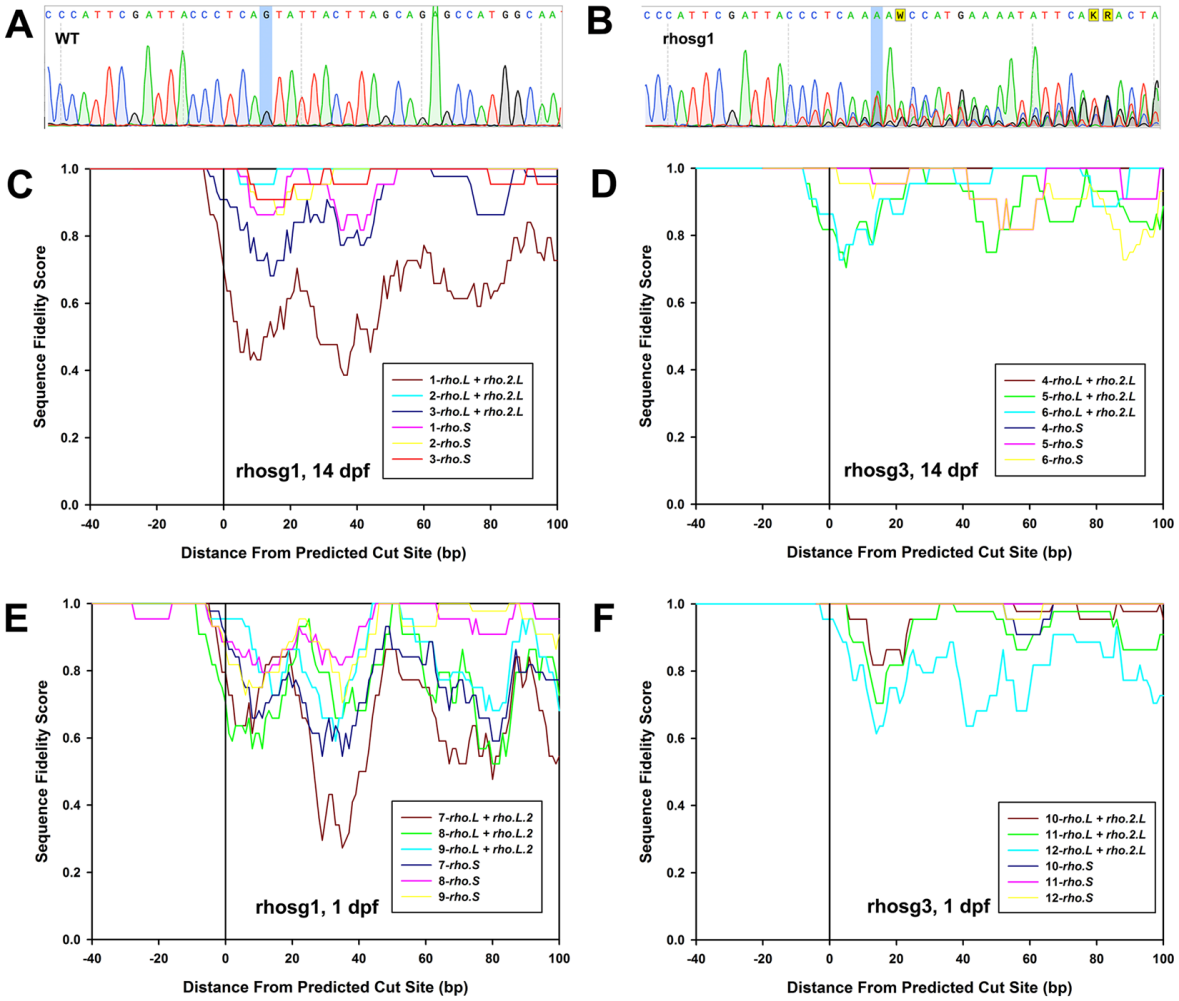
Indels are present in the genomic DNA of F0 animals. (**A,B**) Sample trace reads from dye-termination Sanger sequencing. (**A**) WT sample. (**B**) Edited with rhosg1. Cleavage is predicted to occur after the nucleotide highlighted in blue (numbered “zero” on plots below). (**C**–**F**) Comparisons of gene editing efficiency between guide RNAs, genes, and stage of development. Calculation of the sequence fidelity score is described under Methods. Each plot shows data derived from three animals, numbered 1–3, 4–6, 7–9 and 10–12.

Studies utilizing NHEJ-mediated production of indels typically employ endonuclease-based assays as a rapid means of detecting genome editing, in which PCR products are digested with endonucleases that target mismatched sequences^43^. Due to the presence of the paralogous *rho.L* and *rho.2.L* sequences in our samples that were not distinguished by PCR primers, we found that these assays were unreliable and typically showed digestion of PCR products in WT samples (not shown). Allelic variants in our non-inbred populations of *X. laevis* could also contribute to this effect. Therefore, we adopted an alternate method based on Sanger dye-termination sequencing of PCR products derived from the targeted *rhodopsin* sequences. Whereas WT trace reads typically contain minimal background noise, we found that trace reads derived from animals exhibiting severe phenotypes on rod opsin dot blot assays or confocal microscopy had high levels of noise just prior to and following the predicted cleavage sites (Fig. 3), consistent with a template containing multiple similar sequences of different lengths. We developed a method for comparing samples in which we graphed the moving average of a sequence fidelity score calculated from sequence data files (see methods section for details). This provided a useful means of comparing the extent of genomic DNA editing between samples (Fig. 3C–F). This analysis demonstrated extensive editing of genomic DNA, and also indicated that the majority of indels are small, as the sequence files generally did not show increased miscalling >10 bp prior to the cleavage site.

### Genome editing occurs as early as 1dpf

As a means of determining the approximate timeframe of genomic editing in our embryos, we analyzed genomic DNA from embryos injected with Cas9 mRNA+ rhosg1 or Cas9 mRNA+ rhosg3 at both 14 dpf (developmental stage 48) and 1dpf (developmental stage 12–15) (Nieuwkoop and Faber, 1994). At both developmental stages, many embryos showed extensive editing observed in direct sequencing trace reads (Fig. 3C–F), indicating that editing of the *rhodopsin* genes can occur prior to eye development, at a stage where these genes are not transcriptionally active. Transcriptionally inactive genes may be located in heterochromatin, which Cas9 samples less frequently and scans more slowly, potentially limiting editing by CRISPR/Cas9^44^. The *rhodopsin* genes are not transcriptionally active prior to development of the retina. Based on our results, this transcriptional inactivity does not prevent robust editing of the *X. laevis rhodopsin* genes.

### Confirmation and characterization of indels by sequencing of discrete clones

**Figure 4:**
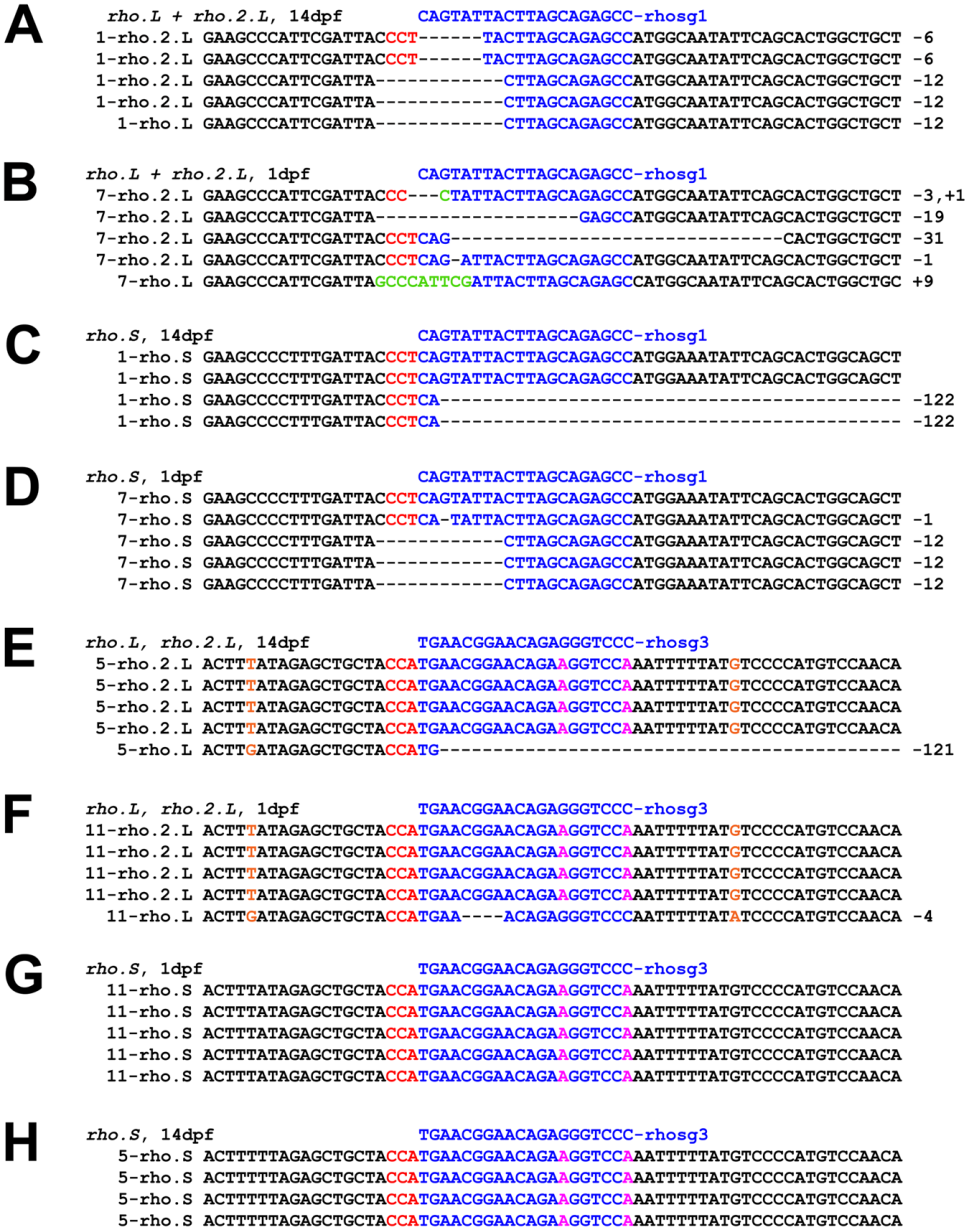
Characterization of indels by sequencing genomic DNA clones: (**A–H**) Sequences obtained from individual pBluescript-SKII+ clones of PCR products derived from genomic DNA isolated from F0 embryos at either 1 dpf or 14 dpf (as indicated) edited with Cas9 mRNA+ rhosg1 or rhosg3 (as indicated). The reverse complement sequence of the guide RNA is shown above the individual genomic DNA sequences in blue. The targeted sequences are also shown in blue. Mismatches with the guide RNA are shown in magenta. Insertions are noted in green. The protospacer adjacent motif (PAM) sequence is shown in red. Bases that are diagnostic of *rho.L* vs. *rho.2.L* are shown in orange. The number at the beginning of each sequence corresponds to sample numbers for direct sequencing results shown in Fig. 3.

To examine individual sequences of the indels generated by these procedures, we cloned PCR products and determined DNA sequences from individual clones. In 1 dpf embryos and 14 dpf tadpoles generated via injection of Cas9 mRNA and rhosg1, we observed indels in all three *rhodopsin* genes (Fig. 4A–D). Small deletions of <20 bp were most commonly observed, but large deletions, including a deletion greater than 100 bp, and small insertions, were also observed, as well as simultaneous deletions and insertions, consistent with results reported for NHEJ-based mutagenesis in other systems^45, 46^ and for editing of *X. laevis tyrosinase* genes^30^. We also noted that identical sequences were sometimes returned in our small samples of clones, suggesting that editing can occur early in development when the number of cells per embryo is low (Fig. 4A). However, in other cases identical clones were not observed, indicating that the timing of editing may be somewhat variable (Fig. 4B), and that the F0 animals are genetic mosaics. Further characterization of indels by techniques such as high-throughput sequencing of multiple F0 embryos would be required to accurately assess the developmental timeframe of genomic editing.

### sgRNAs do not induce indels in mismatched alleles

In 1 dpf embryos and 14 dpf tadpoles generated via injection of Cas9 mRNA and rhosg3, we did not observe significant editing of mismatched *rho.S* and *rho.L* sequences (Fig. 4E–H). In conjunction with the low levels of toxicity observed in animals injected with Cas9 mRNA alone or Cas9 mRNA+ non-targeting rhosgN, this suggests that off-target effects due to non-specific cleavage are likely to be minimal. This result also demonstrates that in an outbred animal model such as *X. laevis*, it may be possible to selectively edit allelic variants in order to specifically generate heterozygous knockouts (potentially extremely useful in cases where homozygous knockout is lethal).

### Germline transmission of edited *rhodopsin* genes

**Figure 5:**
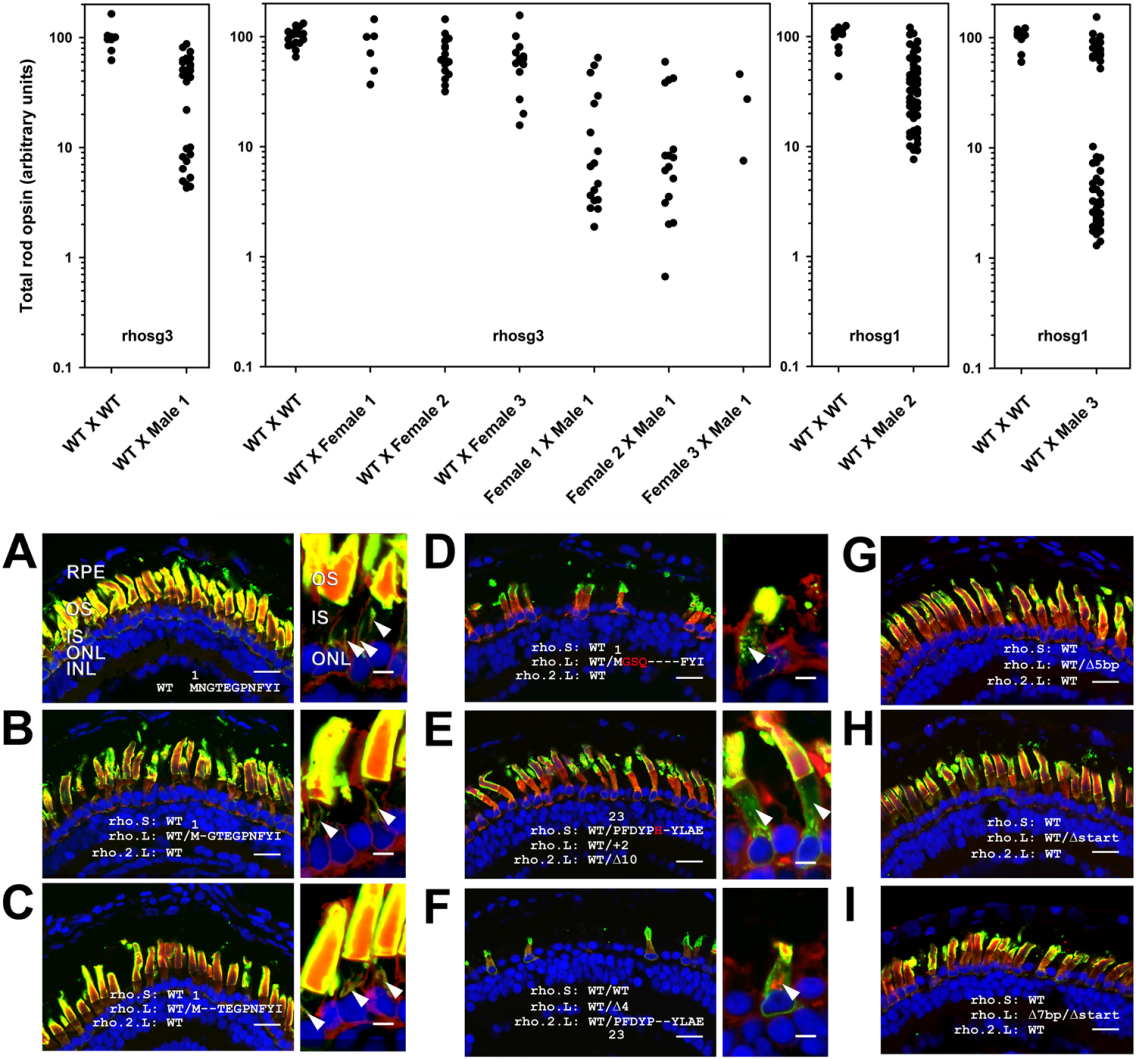
Germline transmission of genomic DNA editing and phenotypes of F1 offspring: Plots: Rod opsin levels assayed in individual eyes by dot blot assay at 14 dpf. Each data point represents a different F1 animal. Each X-axis point represents offspring of a single mating, for which the F0 parents are indicated as being WT and/or F0 animals (named Male 1, Male 2, etc.). Each plot represents samples analyzed on a separate dot blot, and all animals on each plot were modified using the same sgRNA, indicated at the bottom of the plot. (**A**–**I**) Phenotypes of F1 animals assessed by confocal microscopy. (**A**) Wildtype. (**B**–**I)** genetically modified (genotypes indicated on panels, for frame-conserving mutations the altered amino acid sequence is shown, with inserted amino acid residues shown in red). (**B,C**) frame-conserving mutations with minimal phenotype. (**D–F**) frame-conserving indels with significant RD phenotypes. (**G**–**I)** frame shifting indels. (**A–C**) High magnification panels: frame-conserving indels generating minimal or no RD phenotype do not alter rhodopsin localization, which is largely confined to outer segments (OS) and wheat germ agglutinin-positive inner segment (IS) membranes (arrowheads). (**D–F**) High magnification panels: frame-conserving indels generating significant RD phenotypes alter rhodopsin localization in inner segments causing a punctate distribution (**D**, arrowhead) or diffuse labeling consistent with ER retention (**E**,**F**, arrowheads). Green: anti-rhodopsin (B630N – A-I or 514–18 A-D high mag). Red: anti-rod transducin. Blue: Hoechst 33342. Bars = 20 µm (low mag) or 5 µm (high mag). RPE: retinal pigment epithelium. OS: outer segments. IS: inner segments. ONL: outer nuclear layer. INL: inner nuclear layer.

We examined F1 progeny of matings of *X. laevis* derived from embryos injected with rhosg3 or rhosg1 and Cas9 mRNA with either wildtype *X. laevis* or similarly genetically modified animals using techniques similar to those described above. Animals were selected for breeding based on editing observed in genomic DNA isolated from blood samples using analysis similar to Fig. 3 (not shown). Dot blot analysis of total rod opsin levels showed reduced rod opsin in retinal extracts (Fig. 5), and indels were identified by DNA sequencing, indicating successful modification of germline cells in the F0 parents.

### Germline transmission of indels and phenotypes of F1 animals

Dot blot analysis and confocal microscopy indicated the presence of at least two distinct phenotypes in F1 offspring of animals edited with either rhosg3 or rhosg1: moderate reduction of total rod opsin levels (>10% remaining) without significant RD, and severe reduction in total rod opsin levels (<10% remaining) with severe RD (Fig. 5, plots). We performed DNA sequencing analysis on the *rhodopsin* genes in select animals with editing induced by rhosg3 and rhosg1, and recovered both reading frame-conserving and reading frame-shifting indels, as well as indels that resulted in loss of the start codon (Fig. 5A–I). In F1 animals, the severe RD phenotypes were associated with frame-conserving indels in *rho.S, rho.L*, or *rho.2.L*, including indels that replaced seven amino acid residues immediately following the N-terminal methionine of *rho.L* with an unrelated three residue sequence (Fig. 5D), and indels that altered or deleted residues 28 and 29 of *rho.S* or *rho.2.L* (Fig. 5E,F). Frame-conserving indels deleting one or two residues following the start codon of *rho.L* were also recovered, and were not associated with severe RD (Fig. 5B-C). The larger frame-conserving indel in *rho.L* resulted in an abnormal punctate localization of rhodopsin in rod inner segments relative to wildtype or smaller deletions, likely indicating a biosynthetic defect (Fig. 5D), and possibly representing a new class of rhodopsin biosynthetic defects, as a punctate distribution was not previously observed with *rhodopsin* mutants such as P23H, but rather ER retention^16, 47^. The frame conserving indels in *rho.S* and *rho.2.L* were associated with rhodopsin mislocalization more typical of ER retention, in which diffuse inner segment labeling with anti-rhodopsin antibodies became more prominent (Fig. 5E,F).

In contrast, indels that resulted in frameshift or loss of the start codon in *rho.L* were associated with moderate reduction of total rod opsin without apparent RD (Fig. 5G–I), consistent with loss-of-function of one allele of the *rho.L* gene. On crossing two F0 animals, a subset of F1 offspring were recovered with loss-of-function mutations in both *rho.L* alleles; these also showed no obvious signs of rod cell loss, although rod outer segments appeared shorter (Fig. 5I).

Based on these results, it is likely that the phenotypes we observed in genetically mosaic F0 animals (Figs 1 and 2) are due to a combination of loss-of-function frameshifting and gain-of-function frame-conserving mutations, modeling equivalent effects of loss-of-function and gain-of-function rhodopsin mutations responsible for recessive and dominant forms of the human disorder retinitis pigmentosa.

### Phenotypes of *rhodopsin* knockouts (electron microscopy)

**Figure 6:**
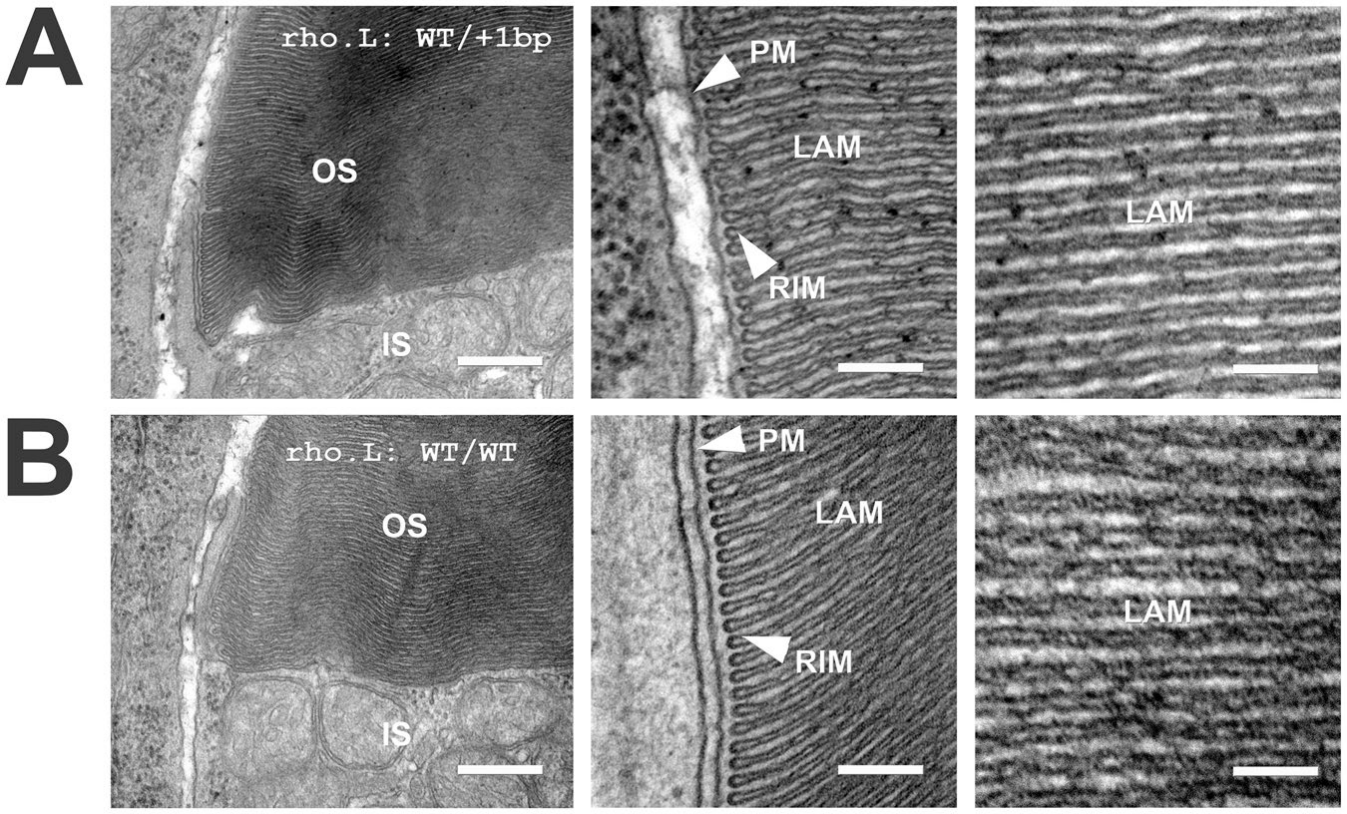
Electron microscopy of rod photoreceptors with loss of function mutations in one *rho.L* allele: (**A**) images of rods with a 1 bp insertion in one *rho.L* allele induced by rhosg3. (**B**) Comparable images from WT rods. Outer segment (OS) disks are densely packed at both the rim and lamellar (LAM) regions. Disk rims are closely apposed to the plasma membrane (PM). No significant abnormalities were identifiable. IS: inner segment. Bar = 400 nm (left) 200 nm (center) or 100 nm (right).

Using transmission electron microscopy (Fig. 6), we examined rod photoreceptors of F1 animals with loss-of-function mutations in one allele of *rho.L* caused by insertion of a single base pair in exon 1 induced by rhosg3. Ultrastructurally, the rod photoreceptors were comparable to wildtype, with regularly spaced and well-organized disk membranes, and closely apposed disk rim and plasma membrane regions, consistent with previous findings of relatively mild phenotypes in heterozygous rhodopsin knockout mice.

### HDR-mediated insertion of a foreign eGFP sequence into *rhodopsin*

**Figure 7:**
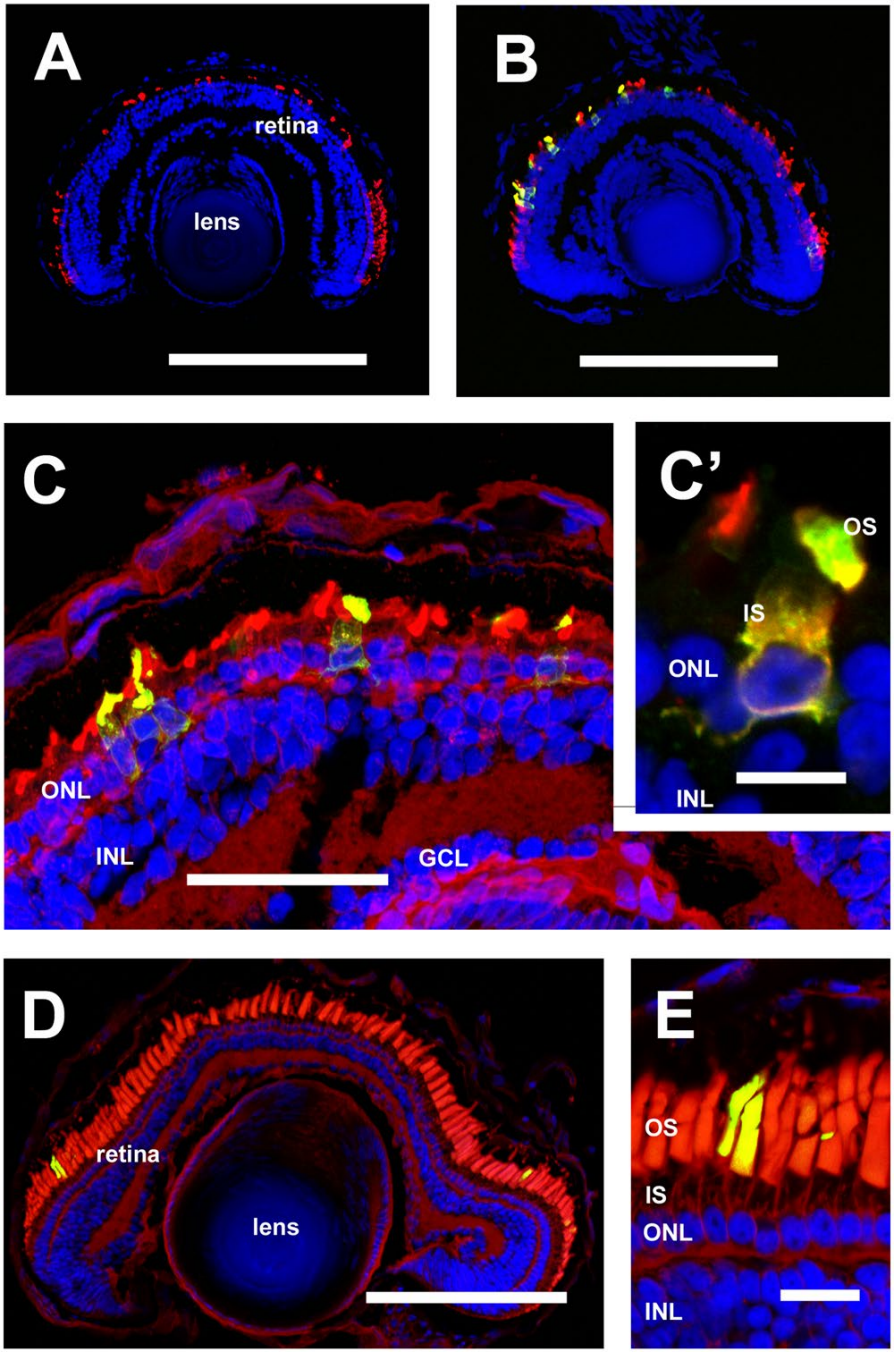
HDR mediated gene alterations assessed by confocal microscopy: (**A**) RD in an animal edited using sgRNA rhosg4 (**B,C,C’**) images derived from an animal in which rhosg4 was used to direct targeted insertion of eGFP into *rho.L*. A number of eGFP-positive rods are present, while other cells of the retina and lens do not show eGFP expression. Significant RD is present (**D,E**) images derived from an animal with targeted mutation of residue M13→F in *rho.L*, and stained with anti-mammalian rhodopsin (2B2). There is no identifiable RD. (**A**–**C**) Green: eGFP. (**D,E**) green: anti-mammalian rhodopsin (2B2). (**A**,**B**) Red: anti-rod transducin. **C**–**E:** Red: wheat germ agglutinin. (**A**–**E**) Blue: Hoechst 33342. OS: outer segments. IS: inner segments. ONL: outer nuclear layer. INL: inner nuclear layer. GCL: ganglion cell layer. (**A**,**B**,**D**) Bar = 200 µm. (**C**) Bar = 50 µm. (**C’**) Bar = 10 µm. (**E**) Bar = 20 µm. Panels C and C’ are confocal projections derived from 10 confocal sections.

To determine whether we could use genome editing by Cas9 mRNA and sgRNAs to induce insertion of foreign DNA sequences by homology-directed repair (HDR) as reported for other systems^32, 48, 49^, we generated a third sgRNA (rhosg4) that targets the fifth exon of all three *rhodopsin* genes, as well as a repair template constructed with approximately 1200 bp of homology to *rho.2.L* around the rhosg4 predicted cut-site, and encoding eGFP at a position in the rhodopsin peptide sequence previously demonstrated to generate a functional rhodopsin-GFP fusion protein^50^. The homology sequence on the repair template also encoded two silent mutations in the rhosg4 recognition sequence to prevent re-cleavage by Cas9 after integration (For complete sequence see Supplemental Figure 2). Injections of Cas9 mRNA and rhosg4 were conducted as described above, with the additional inclusion of the repair template, and eyes of injected tadpoles were screened by fluorescence microscopy during the second week of development. To avoid having eGFP signals originating from two sources, no eGFP mRNA was co-injected, and all injected embryos were screened for the predicted phenotype. eGFP fluorescence was detected in the eyes of two animals out of 456 screened. These were subsequently analyzed by confocal microscopy. Interestingly, both animals showed eGFP expression in both eyes. The eGFP fluorescence was confined to a subset of rod photoreceptors, was highly expressed in outer segments, and was not expressed in other cell types (Fig. 7A–C). The localization was consistent with results previously reported for rhodopsin-GFP fusion proteins expressed in *X. laevis* rods^50–52^. However, these retinas also exhibited significant RD, and rod cells had abnormal morphologies regardless of whether they expressed the fusion, suggesting that homology-directed repair was incomplete. It is possible that this RD reduced the apparent efficiency of the procedure by eliminating fluorescent rods. The RD is likely due to the fact that rhosg4 targets the last exon of all three rhodopsin genes, resulting in gene editing in the absence of nonsense mediated decay and high rates of dominant disease-causing mutations. In agreement, similar retinal degeneration was also observed in retinas of animals edited with rhosg4 in the absence of HDR repair template (Fig. 7A). PCR amplification and Sanger sequencing of the targeted regions of the three *rhodopsin* genes confirmed that eGFP integrated exclusively into *rho.2.L* as predicted, and that rhosg4 also generated indels in the *rho.S* and *rho.L* genes (not shown).

### HDR-mediated insertion of a point mutation into *rhodopsin*

Similarly, we investigated the use of HDR to introduce targeted point mutations, using rhosg1 and a repair template containing approximately 1500 bp of homology to *rho*.2.L flanking the rhosg1 targeting site, and encoding a 2 bp change designed to insert the single amino acid change met13→phe, which was previously demonstrated to confer reactivity to the anti-mammalian rhodopsin antibody 2B2^54, 55^. The repair template similarly encoded two silent mutations designed to eliminate re-cleavage by Cas9 and rhosg1 (Supplemental Figure 2).

We examined retinas of eGFP positive animals using confocal microscopy and 2B2 labeling. Forty-one eyes were cryosectioned, and three were found to contain small numbers of 2B2-positive rods (Fig. 7D,E), while the other eyes did not. The majority of the eyes examined exhibited severe RD, which potentially eliminated 2B2-positive rods, possibly reducing the apparent efficiency of the procedure. One eye containing 2B2-positive rods did not exhibit any RD (Fig. 7D,E); potentially fewer editing events in this animal allowed the genetically modified rods to survive for positive identification.

## Discussion

We have demonstrated manipulation of the genes encoding rhodopsin in the allotetraploid *X. laevis* genome using CRISPR/Cas9 technology, including the generation of knockout and dominant alleles, and targeted insertions. These modifications occur in somatic cells with high efficiency early in development, permitting analysis in F0 animals, and also occur in germline cells, with high efficiency transmission to F1 offspring.

Due to the allotetraploid nature of the *X. laevis* genome, two homeologs are present for most gene products represented by a single gene in diploid species such as humans and mice, and three genes are present for *rhodopsin*. However, based on our experience with *rho.L* and *rho.S*, it is possible to design sgRNAs that will target both *X. laevis* homeologs (Fig. 2), and in the case of *rhodopsin*, a third paralogous gene sequence *rho.2.L*. It is similarly possible to design sgRNAs that will target a single gene (Figs 1 and 4), allowing a researcher to selectively manipulate one or both homeologs, a potentially valuable tool for studying the function of genes that produce lethal phenotypes on complete knockout. Thus, the allotetraploid *X. laevis* genome poses some experimental difficulties relative to genetic manipulation of other species, while also introducing additional versatility.

By injection of mRNA encoding Cas9 and sgRNAs targeting a gene of interest, we were able to generate phenotypes in the majority of F0 animals (Figs 1 and 2). Thus, we are able to perform genetic manipulation experiments in relatively short timeframes, without waiting for F1 offspring. This is similar to paradigms we have used with transgenic *X. laevis*, in which large numbers of F0 transgenic animals are analyzed to obtain insight into gene function^6, 7, 16, 54^. The techniques described here greatly increase the utility of *X. laevis* for experimental paradigms such as disease modeling and investigation of gene function. Additionally, we observed efficient transmission of genetic manipulations to F1 offspring (Fig. 5); thus, interesting experimental results obtained in F0 animals can be confirmed or further characterized at a later date in F1 offspring (Figs 1, 5 and 6).

We noted that in-frame deletions in the *X. laevis rhodopsin* genes can cause dominant phenotypes distinct from the loss-of function obtained with out-of-frame deletions, complicating the analysis of genetically mosaic F0 animals. This is likely due to the fact that rhodopsin is expressed at very high (millimolar) levels in rod outer segment disks^56^, such that defects in biosynthesis and trafficking can cause ER stress and cell death^8, 11, 47, 57–59^. These phenotypes are similar to those observed with missense mutations, and likely model forms of the human disorder retinitis pigmentosa associated with rhodopsin instability^11, 16, 47^. Related phenotypes are less likely to occur with manipulation of genes that are expressed at low levels, where misfolding is unlikely to overload quality control processes and is more likely to cause a variant of loss of function^60^. However, it is possible that unanticipated dominant phenotypes could occur with any gene, as well as relatively innocuous outcomes such as the single and dual amino acid deletions we detected (Fig. 5B,C). Moreover, if gain of function phenotypes are not desired, is likely that phenotypes associated with in-frame deletions could be minimized by injection of multiple sgRNAs targeting multiple exons, increasing the likelihood of obtaining at least one frame-shifting deletion and consequent nonsense-mediated decay. In-frame deletions are also less likely to occur when gene copy numbers are smaller; the presence of six rhodopsin alleles decreases the odds of complete knockout in any given cell due to increased likelihood of in-frame deletions and decreased likelihood of complete editing of all genes. Our results demonstrate that careful examination of the resulting phenotypes is warranted in any experiment of this type.

The phenotypes we observed in animals heterozygous and homozygous for loss-of-function mutations in *rho.2.L* are consistent with phenotypes reported in *rhodopsin* knockout mice, and human carriers of *rhodopsin* mutations responsible for autosomal recessive retinitis pigmentosa^33, 34, 61^. In particular, we observed reduced rod opsin content of retinas, without extensive cell death of rod photoreceptors, and rod outer segments with well-ordered and regularly spaced disks. As in-frame mutations in all three *rhodopsin* genes were associated with RD, our results demonstrate that all three genes are expressed at significant levels. More detailed analysis of F1 offspring will be necessary to establish the exact contributions of each gene, and to determine whether the three genes are expressed consistently throughout development. In addition to modeling the human disorder recessive retinitis pigmentosa, knockout animals will be useful for future examinations of the role of rhodopsin in regulating rod outer segment disk membrane synthesis. Interestingly, we did not observe dramatic alterations in outer segment diameter such as those reported by Makino *et al*. in mice^62–64^, although rod outer segments did appear shorter (Fig. 5H,I).

In addition to introducing simple insertions and deletions via NHEJ, we also attempted to introduce specific point mutations and large coding sequences into the *rho.L* genes via homology directed repair (HDR), including both a point mutation that introduces an antibody epitope and a large sequence encoding GFP, both of which are well characterized from previous use in rhodopsin transgenes^47, 50, 52, 65^. Both types of gene modification were successful, indicating that these techniques could be used to introduce sequences for modeling disease or structure/function studies. However, the desired phenotypes (antibody reactivity, green fluorescence) were not obtained efficiently, although it is possible that the aggressive RD induced by the sgRNAs prevented detection of antibody reactivity or green fluorescence in a significant subset of animals. Thus, unlike analysis of indel-generated phenotypes, it would likely be prohibitively difficult to utilize these approaches as described for routine analysis in F0 animals. However, HDR approaches in F0 animals might be feasible after further optimization. For example, it is likely that HDR efficiency could be improved by treating injected embryos with drugs such as SCR7 that inhibit NHEJ^66, 67^. In addition, targeting a single gene or allele is likely preferable if NHEJ frequently results in dominant cell death phenotypes. Future optimization of HDR in this system would benefit from a sgRNA that targets only a single *rhodopsin* gene in order to reduce the co-occurrence of retinal degeneration. In addition, the use of a shorter single stranded DNA oligonucleotide repair template may improve the efficiency of HDR for introduction of short sequences such as the M13F epitope^53^.

*X. laevis* is an animal frequently used in laboratory investigations, particularly in the fields of developmental biology and vision research. *X. laevis* are valued for the relative ease with which they can be bred, and the rapid *ex-vivo* development of the resulting embryos. Transgene-based genome manipulation strategies have greatly increased the utility of this species as an experimental animal^15^. However, the lack of a robust gene knockout or knockdown strategy was a significant limitation^21^. Our description of efficient knockout strategies using CRISPR/Cas9 will further increase the experimental utility of *X. laevis* in studies examining gene function, including studies of *rhodopsin*. Shared genomics, cell biology, anatomy and physiology between human and *Xenopus* species, along with the increased accessibility of this model through transgenesis and genome editing procedures, make *Xenopus laevis* an exciting and promising candidate for human disease modeling^25^.

## Electronic supplementary material

Supplementary Information
