# Supplementary material for: Modeling Dominant and Recessive Forms of Retinitis Pigmentosa by Editing Three Rhodopsin-Encoding Genes in Xenopus Laevis Using Crispr/Cas9
Source: Sci Rep. 2017 Jul 31;7:6920. doi: 10.1038/s41598-017-07153-4 (PMC5537283; doi:10.1038/s41598-017-07153-4)
Supplement: Supplementary file 1 — Supplementary Information [file 41598_2017_7153_MOESM1_ESM.doc]

**Supplemental Tables and Figures for:**

MODELING DOMINANT AND RECESSIVE FORMS OF RETINITIS PIGMENTOSA BY EDITING THREE *RHODOPSIN*-ENCODING GENES IN *XENOPUS LAEVIS* USING CRISPR/CAS9

**Authors:** Joanna M. Feehan^1,2^, Colette N. Chiu^1^, Paloma Stanar^1^, Beatrice M. Tam^1^, Sheikh N. Ahmed^1^, and Orson L. Moritz^1^

## Corresponding Author:

Orson L. Moritz, Ph.D., Associate Professor

University of British Columbia Dept of Ophthalmology & Visual Sciences

UBC/VGH Eye Care Centre

2550 Willow Street

Vancouver, British Columbia,

Canada

V5Z 3N9

Tel: 604-875-4375

Cell: 604-710-1141

Email: [olmoritz@mail.ubc.ca](mailto:olmoritz@mail.ubc.ca)

**Contents:**

**Supplemental Table 1:** Summary of sequence match scoring procedures for generating the sequence fidelity score: Page 2

**Supplemental Figure 1:** Sequences of *X. laevis rhodopsin* genes, guide RNAs and predicted protein sequences: Page 2

**Supplemental Figure 2:** Plasmid constructs for Homology Directed Repair experiments: Page 8

**Supplemental Table 1 – Summary of sequence match scoring procedure for generating the sequence fidelity score.** Each base in trace read of interest is compared to a WT trace read, and assigned a score using the following matrix. (X = experimental trace read base, W = Wildtype trace read base from equivalent position, X1 or W1 = primary base call, X2 or W2 = secondary base call):

| **Condition** | **Score** |
| --- | --- |
| **X1=W1** | **1** |
| **X1=W1 (both ambiguous, but perfect match, e.g. X1 = R, W1 = R** | **1** |
| **X1 is ambiguous AND X2 = W1** | **0.75** |
| **X1 is ambiguous AND W1 is a partial match (e.g. X1 = R, W1 = G)** | **0.5** |
| **X1 is ambiguous AND W1 is ambiguous AND a partial match (e.g. X1 = R, W1 = S)** | **0.5** |
| **X1 ≠ W1 AND X2 = W1** | **0.25** |
| **X1 ≠ W1 AND X2 ≠ W1** | **0** |

**Supplemental Figure 1 –Sequences of *X. laevis* *rhodopsin* genes, guide RNAs, and predicted protein sequences.**

*rho.L* = XB-GENE-966893

*rho.S* = XB-GENE-17342665

<http://www.xenbase.org/gene/showgene.do?method=display&geneId=XB-GENE-17342665>

*rho.2.L* = XB-GENE-18034123

<http://www.xenbase.org/gene/showgene.do?method=displayGeneSummary&geneId=18034121>

<http://gbrowse.xenbase.org/fgb2/gene_model_details/xl9_1?feature_id=606550>

**sgRNA targeting sequences used in this study:**

rhosg1 (targets *rho.L*, *rho.S*, *rho.2.L*): GGCTCTGCTAAGTAATACTG

Reverse Complement: CAGTATTACTTAGCAGAGCC

(targets first exon)

rhosg3 (targets *rho.2.L*): GGGACCCTCTGTTCCGTTCA

Reverse Complement: TGAACGGAACAGAGGGTCCC

(targets first exon)

rhosg4 (targets *rho.L*, *rho.S*, *rho.2.L*): GGGTGGTGATCAAGCAGTTA

Reverse Complement: TAACTGCTTGATCACCACCC

(targets last exon)

**Nucleotide (coding) sequences (sequences complementary to sgRNA targeting sequences are highlighted):**

>rho.S

ATGAACGGAACAGAAGGTCCAAATTTTTATGTCCCCATGTCCAACAAAACTGGGGTGGTGCGAAGCCCCTTTGATTACCCTCAGTATTACTTAGCAGAGCCATGGAAATATTCAGCACTGGCAGCTTACATGTTCCTGCTCATCCTGCTTGGGTTTCCAATCAACTTCATGACCTTGTATGTCACCATCCAGCACAAGAAACTCAGAACACCCTTAAACTACATCTTGCTGAACCTGGTATTTGCCAATCACTTCATGGTCCTGTGTGGGTTCACGGTAACGATGTACTCCTCAATGCACGGCTACTTCATCTTTGGCCAAACTGGTTGCTACATTGAAGGCTTCTTTGCTACACTCGGTGGTGAAATAGCCCTCTGGTCACTGGTAGTATTGGCCGTTGAAAGATATATGGTGGTCTGCAAGCCCATGGCCAACTTCCGATTTGGGGAGAACCATGCTATTATGGGTGTAATCTTCACATGGATCATGGCTTTGTCTTGTGCTGCTCCTCCTCTCGTTGGATGGTCCAGATACATCCCAGAAGGAATGCAGTGCTCATGTGGAGTAGATTACTATACATTGAAGCCTGAGGTCAACAATGAATCCTTTGTTGTCTACATGTTCATTGTCCACTTCACCATTCCCCTGATTGTCATCTTCTTCTGCTATGGCCGACTGTTGTGCACTGTTAAAGAGGCTGCAGCCCAGCAACAGGAATCTGCTACAACCCAGAAGGCTGAGAAAGAGGTCACCAGAATGGTTATTATCATGGTCGTTTTCTTCTTGATCTGTTGGGTGCCCTATGCCTATGTGGCATTCTACATCTTTACCCACCAGGGCTCTGACTTTGGTCCAGTCTTCATGACCATCCCAGCTTTCTTTGCCAAGAGCTCTTCTATCTACAATCCTGTCATCTACATTCTCTTGAACAAACAGTTCCGTAACTGCTTGATCACCACCCTGTGCTGTGGAAAGAATCCATTCAGTGATGAAGAGGGCTCCTCTGCAGCCACTTCCAAGACAGAAGCTTCTTCTGTCTCTTCCAGCCAGGTGTCTCCTGCATAA

>rho.L

ATGAACGGAACAGAAGGTCCAAATTTTTATGTCCCCATGTCCAACAAAACTGGGGTGGTACGAAGCCCATTCGATTACCCTCAGTATTACTTAGCAGAGCCATGGCAATATTCAGCACTGGCTGCTTACATGTTCCTGCTCATCCTGCTTGGGTTACCAATCAACTTCATGACCTTGTTTGTTACCATCCAGCACAAGAAACTCAGAACACCCCTAAACTACATCCTGCTGAACCTGGTATTTGCCAATCACTTCATGGTCCTGTGTGGGTTCACGGTGACAATGTACACCTCAATGCACGGCTACTTCATCTTTGGCCAAACTGGTTGCTACATTGAAGGCTTCTTTGCTACACTTGGTGGTGAAGTGGCCCTCTGGTCACTGGTAGTATTGGCCGTTGAAAGATATATGGTGGTCTGCAAGCCCATGGCCAACTTCCGATTCGGCGAGAACCATGCTATTATGGGTGTAGCCTTCACATGGATCATGGCTTTGTCTTGTGCTGCTCCTCCTCTCTTCGGATGGTCCAGATACATCCCAGAGGGAATGCAATGCTCATGCGGAGTAGACTACTACACACTGAAGCCTGAGGTCAACAATGAATCCTTTGTTATCTACATGTTCATTGTCCACTTCACCATTCCCCTGATTGTCATCTTCTTCTGCTATGGTCGCCTGCTCTGCACTGTCAAAGAGGCTGCAGCCCAGCAACAGGAATCTGCTACCACCCAGAAGGCTGAGAAAGAGGTCACCAGAATGGTTGTTATCATGGTCGTTTTCTTCCTGATCTGTTGGGTGCCCTATGCCTATGTGGCATTCTACATCTTCACCCACCAGGGCTCTAACTTTGGCCCAGTCTTCATGACCGTCCCAGCTTTCTTTGCCAAGAGCTCTGCTATCTACAATCCTGTCATCTACATTGTCTTGAACAAACAGTTCCGTAACTGCTTGATCACCACCCTATGCTGTGGAAAGAATCCATTCGGTGATGAAGATGGCTCCTCTGCAGCCACCTCCAAGACAGAAGCTTCTTCTGTCTCTTCCAGCCAGGTGTCTCCTGCATAA

>rho.2.L

ATGAACGGAACAGAGGGTCCCAATTTTTATATCCCCATGTCCAACAAAACTGGGGTGGTACGAAGCCCATTCGATTACCCTCAGTATTACTTAGCAGAGCCATGGCAATATTCAGCACTGGCTGCTTACATGTTCCTGCTCATCCTGCTTGGGTTACCAATCAACTTCATGACCTTGTTTGTTACCATCCAGCACAAGAAACTCAGAACACCCCTAAACTACATCCTGCTGAACCTGGTATTTGCCAATCACTTCATGGTCCTGTGTGGGTTCACGGTGACAATGTACACCTCAATGCACGGCTACTTCATCTTTGGCCAAACTGGTTGCTACATTGAAGGCTTCTTTGCTACACTTGGTGGTGAAGTGGCCCTCTGGTCACTGGTAGTATTGGCCGTTGAAAGATATATGGTGGTCTGCAAGCCCATGGCCAACTTCCGATTCGGGGAGAACCATGCTATCATGGGTGTAGCCTTCACATGGATCATGGCTTTGTCTTGTGCTGCTCCTCCTCTATTTGGATGGTCCAGATACATCCCAGAGGGAATGCAATGCTCATGCGGAGTAGACTACTACACACTGAAGCCTGAGGTCAACAATGAATCCTTTGTTATCTACATGTTCGTTGTCCACTTCACCATTCCCCTGATTGTCATCTTCTTCTGCTATGGCCGCCTGCTCTGCACTGTCAAAGAGGCTGCAGCCCAGCAACAGGAATCTGCTACCACCCAGAAGGCTGAGAAAGAGGTCACCAGAATGGTTGTTATCATGGTCGTTTTCTTCCTGATCTGTTGGGTGCCCTATGCCTATGTGGCATTCTACATCTTCACCCACCAGGGCTCCGACTTTGGCCCAGTCTTCATGACTGTCCCAGCTTTCTTTGCCAAGAGCTCTGCTATCTACAATCCTGTCATCTACATTGTTTTGAACAAACAGTTCCGTAACTGCTTGATCACCACCCTGTGCTGTGGAAAGAATCCATTCGGTGATGAAGATGGCTCCTCTGCAGCCACCTCCAAGACAGAAGCTTCTTCTGTCTCTTCCAGCCAGGTGTCTCCTGCATAA

**Nucleotide (coding sequence) alignment (sequences complementary to guide RNAs highlighted):**

rho.S ATGAACGGAACAGAAGGTCCAAATTTTTATGTCCCCATGTCCAACAAAACTGGGGTGGTG

rho.L ATGAACGGAACAGAAGGTCCAAATTTTTATGTCCCCATGTCCAACAAAACTGGGGTGGTA

rho.2.L ATGAACGGAACAGAGGGTCCCAATTTTTATATCCCCATGTCCAACAAAACTGGGGTGGTA

**************.*****.*********.****************************.

rho.S CGAAGCCCCTTTGATTACCCTCAGTATTACTTAGCAGAGCCATGGAAATATTCAGCACTG

rho.L CGAAGCCCATTCGATTACCCTCAGTATTACTTAGCAGAGCCATGGCAATATTCAGCACTG

rho.2.L CGAAGCCCATTCGATTACCCTCAGTATTACTTAGCAGAGCCATGGCAATATTCAGCACTG

********.** *********************************.**************

rho.S GCAGCTTACATGTTCCTGCTCATCCTGCTTGGGTTTCCAATCAACTTCATGACCTTGTAT

rho.L GCTGCTTACATGTTCCTGCTCATCCTGCTTGGGTTACCAATCAACTTCATGACCTTGTTT

rho.2.L GCTGCTTACATGTTCCTGCTCATCCTGCTTGGGTTACCAATCAACTTCATGACCTTGTTT

**:********************************:**********************:*

rho.S GTCACCATCCAGCACAAGAAACTCAGAACACCCTTAAACTACATCTTGCTGAACCTGGTA

rho.L GTTACCATCCAGCACAAGAAACTCAGAACACCCCTAAACTACATCCTGCTGAACCTGGTA

rho.2.L GTTACCATCCAGCACAAGAAACTCAGAACACCCCTAAACTACATCCTGCTGAACCTGGTA

** ****************************** *********** **************

rho.S TTTGCCAATCACTTCATGGTCCTGTGTGGGTTCACGGTAACGATGTACTCCTCAATGCAC

rho.L TTTGCCAATCACTTCATGGTCCTGTGTGGGTTCACGGTGACAATGTACACCTCAATGCAC

rho.2.L TTTGCCAATCACTTCATGGTCCTGTGTGGGTTCACGGTGACAATGTACACCTCAATGCAC

**************************************.**.******:***********

rho.S GGCTACTTCATCTTTGGCCAAACTGGTTGCTACATTGAAGGCTTCTTTGCTACACTCGGT

rho.L GGCTACTTCATCTTTGGCCAAACTGGTTGCTACATTGAAGGCTTCTTTGCTACACTTGGT

rho.2.L GGCTACTTCATCTTTGGCCAAACTGGTTGCTACATTGAAGGCTTCTTTGCTACACTTGGT

******************************************************** ***

rho.S GGTGAAATAGCCCTCTGGTCACTGGTAGTATTGGCCGTTGAAAGATATATGGTGGTCTGC

rho.L GGTGAAGTGGCCCTCTGGTCACTGGTAGTATTGGCCGTTGAAAGATATATGGTGGTCTGC

rho.2.L GGTGAAGTGGCCCTCTGGTCACTGGTAGTATTGGCCGTTGAAAGATATATGGTGGTCTGC

******.*.***************************************************

rho.S AAGCCCATGGCCAACTTCCGATTTGGGGAGAACCATGCTATTATGGGTGTAATCTTCACA

rho.L AAGCCCATGGCCAACTTCCGATTCGGCGAGAACCATGCTATTATGGGTGTAGCCTTCACA

rho.2.L AAGCCCATGGCCAACTTCCGATTCGGGGAGAACCATGCTATCATGGGTGTAGCCTTCACA

*********************** ** ************** *********. *******

rho.S TGGATCATGGCTTTGTCTTGTGCTGCTCCTCCTCTCGTTGGATGGTCCAGATACATCCCA

rho.L TGGATCATGGCTTTGTCTTGTGCTGCTCCTCCTCTCTTCGGATGGTCCAGATACATCCCA

rho.2.L TGGATCATGGCTTTGTCTTGTGCTGCTCCTCCTCTATTTGGATGGTCCAGATACATCCCA

***********************************. * *********************

rho.S GAAGGAATGCAGTGCTCATGTGGAGTAGATTACTATACATTGAAGCCTGAGGTCAACAAT

rho.L GAGGGAATGCAATGCTCATGCGGAGTAGACTACTACACACTGAAGCCTGAGGTCAACAAT

rho.2.L GAGGGAATGCAATGCTCATGCGGAGTAGACTACTACACACTGAAGCCTGAGGTCAACAAT

**.********.******** ******** ***** *** ********************

rho.S GAATCCTTTGTTGTCTACATGTTCATTGTCCACTTCACCATTCCCCTGATTGTCATCTTC

rho.L GAATCCTTTGTTATCTACATGTTCATTGTCCACTTCACCATTCCCCTGATTGTCATCTTC

rho.2.L GAATCCTTTGTTATCTACATGTTCGTTGTCCACTTCACCATTCCCCTGATTGTCATCTTC

************.***********.***********************************

rho.S TTCTGCTATGGCCGACTGTTGTGCACTGTTAAAGAGGCTGCAGCCCAGCAACAGGAATCT

rho.L TTCTGCTATGGTCGCCTGCTCTGCACTGTCAAAGAGGCTGCAGCCCAGCAACAGGAATCT

rho.2.L TTCTGCTATGGCCGCCTGCTCTGCACTGTCAAAGAGGCTGCAGCCCAGCAACAGGAATCT

*********** **.*** * ******** ******************************

rho.S GCTACAACCCAGAAGGCTGAGAAAGAGGTCACCAGAATGGTTATTATCATGGTCGTTTTC

rho.L GCTACCACCCAGAAGGCTGAGAAAGAGGTCACCAGAATGGTTGTTATCATGGTCGTTTTC

rho.2.L GCTACCACCCAGAAGGCTGAGAAAGAGGTCACCAGAATGGTTGTTATCATGGTCGTTTTC

*****.************************************.*****************

rho.S TTCTTGATCTGTTGGGTGCCCTATGCCTATGTGGCATTCTACATCTTTACCCACCAGGGC

rho.L TTCCTGATCTGTTGGGTGCCCTATGCCTATGTGGCATTCTACATCTTCACCCACCAGGGC

rho.2.L TTCCTGATCTGTTGGGTGCCCTATGCCTATGTGGCATTCTACATCTTCACCCACCAGGGC

*** ******************************************* ************

rho.S TCTGACTTTGGTCCAGTCTTCATGACCATCCCAGCTTTCTTTGCCAAGAGCTCTTCTATC

rho.L TCTAACTTTGGCCCAGTCTTCATGACCGTCCCAGCTTTCTTTGCCAAGAGCTCTGCTATC

rho.2.L TCCGACTTTGGCCCAGTCTTCATGACTGTCCCAGCTTTCTTTGCCAAGAGCTCTGCTATC

** .******* ************** .************************** *****

rho.S TACAATCCTGTCATCTACATTCTCTTGAACAAACAGTTCCGTAACTGCTTGATCACCACC

rho.L TACAATCCTGTCATCTACATTGTCTTGAACAAACAGTTCCGTAACTGCTTGATCACCACC

rho.2.L TACAATCCTGTCATCTACATTGTTTTGAACAAACAGTTCCGTAACTGCTTGATCACCACC

********************* * ************************************

rho.S CTGTGCTGTGGAAAGAATCCATTCAGTGATGAAGAGGGCTCCTCTGCAGCCACTTCCAAG

rho.L CTATGCTGTGGAAAGAATCCATTCGGTGATGAAGATGGCTCCTCTGCAGCCACCTCCAAG

rho.2.L CTGTGCTGTGGAAAGAATCCATTCGGTGATGAAGATGGCTCCTCTGCAGCCACCTCCAAG

**.*********************.********** ***************** ******

rho.S ACAGAAGCTTCTTCTGTCTCTTCCAGCCAGGTGTCTCCTGCATAA

rho.L ACAGAAGCTTCTTCTGTCTCTTCCAGCCAGGTGTCTCCTGCATAA

rho.2.L ACAGAAGCTTCTTCTGTCTCTTCCAGCCAGGTGTCTCCTGCATAA

*********************************************

**Predicted peptide sequences**

>*rho.S*

MNGTEGPNFYVPMSNKTGVVRSPFDYPQYYLAEPWKYSALAAYMFLLILLGFPINFMTLYVTIQHKKLRTPLNYILLNLVFANHFMVLCGFTVTMYSSMHGYFIFGQTGCYIEGFFATLGGEIALWSLVVLAVERYMVVCKPMANFRFGENHAIMGVIFTWIMALSCAAPPLVGWSRYIPEGMQCSCGVDYYTLKPEVNNESFVVYMFIVHFTIPLIVIFFCYGRLLCTVKEAAAQQQESATTQKAEKEVTRMVIIMVVFFLICWVPYAYVAFYIFTHQGSDFGPVFMTIPAFFAKSSSIYNPVIYILLNKQFRNCLITTLCCGKNPFSDEEGSSAATSKTEASSVSSSQVSPA

>*rho.L*

MNGTEGPNFYVPMSNKTGVVRSPFDYPQYYLAEPWQYSALAAYMFLLILLGLPINFMTLFVTIQHKKLRTPLNYILLNLVFANHFMVLCGFTVTMYTSMHGYFIFGQTGCYIEGFFATLGGEVALWSLVVLAVERYMVVCKPMANFRFGENHAIMGVAFTWIMALSCAAPPLFGWSRYIPEGMQCSCGVDYYTLKPEVNNESFVIYMFIVHFTIPLIVIFFCYGRLLCTVKEAAAQQQESATTQKAEKEVTRMVVIMVVFFLICWVPYAYVAFYIFTHQGSNFGPVFMTVPAFFAKSSAIYNPVIYIVLNKQFRNCLITTLCCGKNPFGDEDGSSAATSKTEASSVSSSQVSPA

>*rho.2.L*

MNGTEGPNFYIPMSNKTGVVRSPFDYPQYYLAEPWQYSALAAYMFLLILLGLPINFMTLFVTIQHKKLRTPLNYILLNLVFANHFMVLCGFTVTMYTSMHGYFIFGQTGCYIEGFFATLGGEVALWSLVVLAVERYMVVCKPMANFRFGENHAIMGVAFTWIMALSCAAPPLFGWSRYIPEGMQCSCGVDYYTLKPEVNNESFVIYMFVVHFTIPLIVIFFCYGRLLCTVKEAAAQQQESATTQKAEKEVTRMVVIMVVFFLICWVPYAYVAFYIFTHQGSDFGPVFMTVPAFFAKSSAIYNPVIYIVLNKQFRNCLITTLCCGKNPFGDEDGSSAATSKTEASSVSSSQVSPA

**Predicted peptide sequence alignment**

rho.S MNGTEGPNFYVPMSNKTGVVRSPFDYPQYYLAEPWKYSALAAYMFLLILLGFPINFMTLY

rho.L MNGTEGPNFYVPMSNKTGVVRSPFDYPQYYLAEPWQYSALAAYMFLLILLGLPINFMTLF

rho.2.L MNGTEGPNFYIPMSNKTGVVRSPFDYPQYYLAEPWQYSALAAYMFLLILLGLPINFMTLF

**********:************************:***************:*******:

rho.S VTIQHKKLRTPLNYILLNLVFANHFMVLCGFTVTMYSSMHGYFIFGQTGCYIEGFFATLG

rho.L VTIQHKKLRTPLNYILLNLVFANHFMVLCGFTVTMYTSMHGYFIFGQTGCYIEGFFATLG

rho.2.L VTIQHKKLRTPLNYILLNLVFANHFMVLCGFTVTMYTSMHGYFIFGQTGCYIEGFFATLG

************************************:***********************

rho.S GEIALWSLVVLAVERYMVVCKPMANFRFGENHAIMGVIFTWIMALSCAAPPLVGWSRYIP

rho.L GEVALWSLVVLAVERYMVVCKPMANFRFGENHAIMGVAFTWIMALSCAAPPLFGWSRYIP

rho.2.L GEVALWSLVVLAVERYMVVCKPMANFRFGENHAIMGVAFTWIMALSCAAPPLFGWSRYIP

**:********************************** **************.*******

rho.S EGMQCSCGVDYYTLKPEVNNESFVVYMFIVHFTIPLIVIFFCYGRLLCTVKEAAAQQQES

rho.L EGMQCSCGVDYYTLKPEVNNESFVIYMFIVHFTIPLIVIFFCYGRLLCTVKEAAAQQQES

rho.2.L EGMQCSCGVDYYTLKPEVNNESFVIYMFVVHFTIPLIVIFFCYGRLLCTVKEAAAQQQES

************************:***:*******************************

rho.S ATTQKAEKEVTRMVIIMVVFFLICWVPYAYVAFYIFTHQGSDFGPVFMTIPAFFAKSSSI

rho.L ATTQKAEKEVTRMVVIMVVFFLICWVPYAYVAFYIFTHQGSNFGPVFMTVPAFFAKSSAI

rho.2.L ATTQKAEKEVTRMVVIMVVFFLICWVPYAYVAFYIFTHQGSDFGPVFMTVPAFFAKSSAI

**************:**************************:*******:********:*

rho.S YNPVIYILLNKQFRNCLITTLCCGKNPFSDEEGSSAATSKTEASSVSSSQVSPA

rho.L YNPVIYIVLNKQFRNCLITTLCCGKNPFGDEDGSSAATSKTEASSVSSSQVSPA

rho.2.L YNPVIYIVLNKQFRNCLITTLCCGKNPFGDEDGSSAATSKTEASSVSSSQVSPA

*******:********************.**:**********************

**Supplemental Figure 2: Plasmid constructs for Homology Directed Repair experiments**

CCACCAGTCTACAGCTGTCATCTAGGAATGGTGGAAGTTTCAGTTCAACCAAAGCAAAGAGTGCTAAACATGAGCTGATTAACTATGACTCCCATTGTCTATAGCTGCAGTTAAAAAAAGTCATGGAGAATGTTCTGCTACTTATGATGACTGTGTTGCCTTAGGATCAGCATACAACTTGGGGTAGTAAGGGCATTTGTCTTAGGTCCCAAAATGGAAGGGGCTTTTAGAGGAGAAACACAGATAAAACTACAAAGACCACCAGTGTTTTAGTATTTTAAAACACGGGTAATAACCCTTGAGCAAACTTCGTGTGCTGGATTAATTATTACGTACCTTTCGTTGGGGGTGCCGTCCTCCTATGATCCATGCACCGTGAACGTTGAAAGAGGGACCAGGTGTGCATCTGCATTTCGTTTGGCTTAGTATTTTACAACCTTCGTATACATCAATATGCAATGCATTACTTATTTTTAGGGTAGTAGAGACTAAATGAGTACAACAATACAGAAGAGATGAATGGACTAGATAATGACTGTTCTCCTTTCTCTCCCCAGTTCCGTAA**T**TG**T**TTGATCACCACCCTGTGCTGTGGAAAGAATCCATTCGGTGATGAAGATGGCTCCTCTATGGTGAGCAAGGGCGAGGAGCTGTTCACCGGGGTGGTGCCCATCCTGGTCGAGCTGGACGGCGACGTAAACGGCCACAAGTTCAGCGTGTCCGGCGAGGGCGAGGGCGATGCCACCTACGGCAAGCTGACCCTGAAGTTCATCTGCACCACCGGCAAGCTGCCCGTGCCCTGGCCCACCCTCGTGACCACCCTGACCTACGGCGTGCAGTGCTTCAGCCGCTACCCCGACCACATGAAGCAGCACGACTTCTTCAAGTCCGCCATGCCCGAAGGCTACGTCCAGGAGCGCACCATCTTCTTCAAGGACGACGGCAACTACAAGACCCGCGCCGAGGTGAAGTTCGAGGGCGACACCCTGGTGAACCGCATCGAGCTGAAGGGCATCGACTTCAAGGAGGACGGCAACATCCTGGGGCACAAGCTGGAGTACAACTACAACAGCCACAACGTCTATATCATGGCCGACAAGCAGAAGAACGGCATCAAGGTGAACTTCAAGATCCGCCACAACATCGAGGACGGCAGCGTGCAGCTCGCCGACCACTACCAGCAGAACACCCCCATCGGCGACGGCCCCGTGCTGCTGCCCGACAACCACTACCTGAGCACCCAGTCCGCCCTGAGCAAAGACCCCAACGAGAAGCGCGATCACATGGTCCTGCTGGAGTTCGTGACCGCCGCCGGGATCACTCTCGGCATGGACGAGCTGTACAAGGCAGCCACCTCCAAGACAGAAGCTTCTTCTGTCTCTTCCAGCCAGGTGTCTCCTGCATAAGAGCTTCACCAGGGCTGTCTCAGGGTCCGCTGCCTCACACAATTCCCATCACTTAAGCCCTGTCTACTTGTTGCGAAGGCAAAGAATTCCACAGTTTTAATATTTACCCCCATTCTGCCCAACCTTGGACACTGTAAGAGCTGACCCCATTACTGCTGGGAAGGCCCAAGCTTTGTTGCATTCTGATGTGATCCTTTCAGCAGAAAATGGGTGGATTCAATGAATTTCACCAAGGCTGTACATAACAATAACATTAGTCTGAAGGCACCTCCCACCCAGAGAATGCAACACTTATTTATCTCTGTCTTTTCTTGACATATTGATGCTGCTTCTATTCATGGTCACTAACAAAAAGTCCCATTTTACAATGCAACTGAAAGTAATGTATTTTTGTAATATAATAACATATTTCATGCAATCTCCTCTGCTTATTGGCAAGGTCTGATATAGTGAGGATAGACAGCCAGACCC

## Above: HDR Construct Design for introduction of eGFP.

The 1874 bp sequence above, derived from *rho.2.L* and eGFP, was cloned into the EcoRV site of pbluescript-SKII+. Highlighted sequences are *rho.2.L exon 5 and noncoding sequence*, eGFP, the *rho.2.L* stop codon, the rhosg4 target site, and silent target site mutations.

GTGGCTTATGGGTTAAAAAGGTGCAACACAAACAAATAATCTATTATTTACACACTAGTCAAGACTGGTGCTCAGCTGTGGTTTGAAGATTCTAATTCAATGAACTAATGGTAACCAGGGCCGGATTTGTATTTCTGCAGCCCCTAGGCCATGCGGTCCTAACGTCTGTCCACGACGAGTCTTATTGCCATCCACCCGCAACTCCCGCAAGTGCAAATTTTGGAGCACTGGTGCTCTTCAGCAAGTGGCTGGGCGGCATGCCGTCCCTAAAAGTTCGCCGCCCTAGGCACAGGCCTTTGTGGCCTCTCCACAAATCCAAGCCTGATGGTAACTAAATGTAGAGGGAACTGAGTAAACCCCAAAAATGGCTGCCCTGGCTCCTACAATATGGAATTATCTCCTGTAGGTCAGACCTGGATTTCTTCCTGTCACTTTTAAATACACTTTCTTCTTGTGTGTTTAACAGAGAGAGAGATTGACAGGTGTAGACTTAATACGTTTAAGGGAAGCCAATTAACACTTTGCAATTTTAGCTTGGATTACAGTGATTAATAGTGCGCTAAATCCTTTGTTGCTGACGCTGGGGGTTGCAAGCTTACTCCAGGTGGGACTTTAAAAGGACGAGGGGACAGTGGGTCATACTGTAGAACAGCTTCAGTTGGGATCACAGGCTTCTAGGGATCCTTTGGGCAAAAAAGAAACACAGAAGGCATTCTTTCTATACAAGAAAGGACTTTATAGAGCTGCTACCATGAACGGAACAGAAGGTCCAAATTTTTATGTCCCC**TTT**TCCAACAAAACTGGGGTGGTACGAAGCCCATTCGATTACCCTCA**A**TA**C**TACTTAGCAGAGCCATGGCAATATTCAGCACTGGCTGCTTACATGTTCCTGCTCATCCTGCTTGGGTTACCAATCAACTTCATGACCTTGTTTGTTACCATCCAGCACAAGAAACTCAGAACACCCCTAAACTACATCCTGCTGAACCTGGTATTTGCCAATCACTTCATGGTCCTGTGTGGGTTCACGGTGACAATGTACACCTCAATGCACGGCTACTTCATCTTTGGCCAAACTGGTTGCTACATTGAAGGCTTCTTTGCTACACTTGGTGGTAAGTTCCAATGGGCTTTCGTCACTGATATTGTTGTAGCAATAAATTCTTGGAAAGCTCGTAAGGGAACAAGCTACCAGGGAAAGGGTTATAGGGCTGAAAAGGAATATCAGTACTTTCTATGTTCTCCAGCAGAGTGTAGTGCATACCATGTTAGAGAAAGTTCAACATGTAATACTGTGAGGGGCCAAATTGCCTGGGTTGAGTCATGTTAACCTTTTGCCTTTTCCTGTTCTTTTTACTAACAGGTGAAGTGGCCCTCTGGTCACTGGTAGTATTGGCCGTTGAAAGATATATGGTGGTCTGCAAGCCCATGGCCAACTTCCGATTCGGGGAGAACCATGCTATTATGGGTGTAGCCTTCACATGGATCATGGCTTTGTCTTGTGCTGCTCCTCCTCTCTTCGGATGGTCCAGGTAAATATATATAACATCAGTCAGCATATCCCTAG

## Above: HDR Construct Design for introduction of the met13🡪phe mutation.

The 1564 bp sequence above, derived from *rho.2.L*, was cloned into the EcoRV site of pbluescript-SKII+. Highlighted sequences are *rho.2.L exon 1 and upstream sequence*, the *rho.2.L* start codon, the met13🡪phe mutation, the rhosg1 target site, and silent target site mutations.
